# A Game of Life with dormancy

**DOI:** 10.1098/rspb.2024.2543

**Published:** 2025-01-29

**Authors:** Daniel Henrik Nevermann, Claudius Gros, Jay T. Lennon

**Affiliations:** ^1^ Institute for Theoretical Physics, Goethe-Universitat Frankfurt, Frankfurt, Germany; ^2^ Department of Biology, Indiana University, Bloomington, IN 47405, USA

**Keywords:** complexity, extinction, seed bank, longevity, stochastic, cellular automaton

## Abstract

The factors contributing to the persistence and stability of life are fundamental for understanding complex living systems. Organisms are commonly challenged by harsh and fluctuating environments that are suboptimal for growth and reproduction, which can lead to extinction. Many species contend with unfavourable and noisy conditions by entering a reversible state of reduced metabolic activity, a phenomenon known as dormancy. Here, we develop Spore Life, a model to investigate the effects of dormancy on population dynamics. It is based on Conway’s Game of Life (GoL), a deterministic cellular automaton where simple rules govern the metabolic state of an individual based on the metabolic state of its neighbours. For individuals that would otherwise die, Spore Life provides a refuge in the form of an inactive state. These dormant individuals (spores) can resuscitate when local conditions improve. The model includes a parameter α∈[0,1] that controls the survival probability of spores, interpolating between GoL (α=0) and Spore Life (α=1), while capturing stochastic dynamics in the intermediate regime (0<α<1). In addition to identifying the emergence of unique periodic configurations, we find that spore survival increases the average number of active individuals and buffers populations from extinction. Contrary to expectations, stabilization of the population is not the result of a large and long-lived seed bank. Instead, the demographic patterns in Spore Life only require a small number of resuscitation events. Our approach yields novel insight into what is minimally required for the origins of complex behaviours associated with dormancy and the seed banks that they generate.

## 1. Introduction

In nature, organisms are often challenged by conditions that are suboptimal for growth and reproduction. For life to persist, organisms must contend with a range of external forces. Scarcity of resources, fluctuating abiotic variables, and the patchy distribution of suitable habitats are just a few of the many exogenous factors that can reduce organismal fitness. In addition, individual performance is affected by endogenous factors arising from stochastic demographic events and species interactions like competition and predation. Together, these factors increase the risk of local extinction. Populations can escape this fate through behavioural modifications, phenotypic plasticity, migration within a landscape and evolutionary adaptation [1,2].

One process that is important in promoting the persistence of populations is dormancy, which occurs when an individual enters a reversible state of reduced metabolic activity [3–5]. Dormant individuals enjoy protection against unfavourable conditions and have the capacity to resume growth when conditions improve. The accumulation of inactive individuals results in a ‘seed bank’, which can buffer population dynamics. This buffering can lead to increased geometric mean fitness and a reduced probability of stochastic extinction in variable environments [6,7].

Dormancy is achieved among species in different ways. In some cases, dormancy requires hundreds of interacting genes that are integrated into a tightly regulated developmental program [8]. Organisms adopting such a strategy often rely on the interpretation of environmental cues to transition between metabolic states in a responsive manner [9]. In other cases, dormancy is achieved through simpler means. For example, organisms can stochastically fall into an inactive state or randomly awaken independently of environmental conditions consistent with a bet-hedging strategy [10,11]. These fundamental differences in dormancy affect the size and longevity of a seed bank. While individuals belonging to some species only remain dormant for a short period of time [12], individuals belonging to other species can persist indefinitely [13]. Such differences in shallow versus deep dormancy should have implications for population stability and the persistence of life.

Dormancy has independently arisen many times across the tree of life [7]. In this way, it is an example of convergent evolution [14], suggesting that dormancy may be a solution to one of life’s major problems, i.e. persisting in noisy and unpredictable environments. Despite its prevalence among diverse lineages and ecosystems, there is no standard way to model dormancy. While dynamical approaches have been developed [15–17], they typically do not capture the fact that dormancy is an individual process that can lead to complex emergent behaviours and patterns. Furthermore, modelling efforts have not identified the minimal requirements for achieving the benefits of dormancy, which could shed light on the origins and evolution of an important life-history strategy [18].

One way to model living systems is through a bottom-up approach where rules are encoded into individual agents that are then observed as the consequences unfold over time. Perhaps the best examples come from cellular automata where self-replicating behaviours are governed by the agent’s state (e.g. alive versus dead) as well as those of its neighbours on a two-dimensional lattice with a random set of initial conditions. Despite their simplicity at the agent level, cellular automata, most notably Conway’s Game of Life (GoL) [19], can produce periodic patterns [20], chaotic dynamics [21], self-organized criticality [22] and other life-like complexities [23]. While dispersal in space has been explored in cellular automata [24,25], few studies have investigated how dispersal in time (i.e. dormancy) affects the dynamics of such systems [26,27].

Here, we develop a cellular automaton called Spore Life, which includes minimal modifications to the original GoL. We quantify the distribution of metabolic states along with the probability of extinction with updated rules that capture the core features of dormancy. In particular, we explored shallow versus deep dormancy by manipulating a spore survivorship parameter, which also introduces stochasticity into the model. By characterizing the lifetime of individuals in active (A) and inactive (I) states, along with spore effects on demographic rates (i.e. births and deaths), we are able to recreate and better understand what is minimally required for dormancy and its contribution to population persistence.

## 2. Methods

### (a) Model description

**Figure 1.**
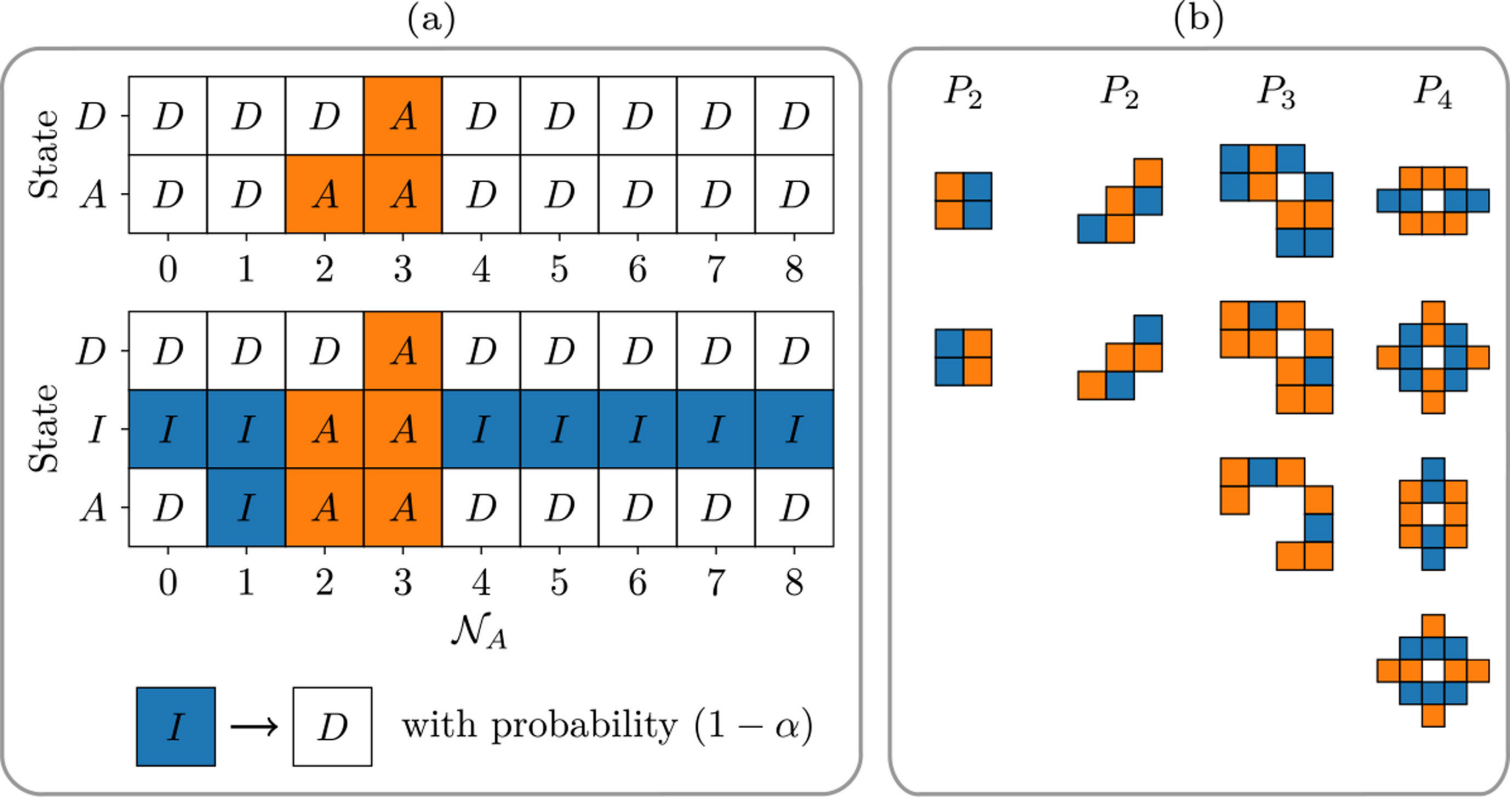
Transition rules and periodic configurations for a cellular automaton with dormancy. In (*a*) rule tables for the GoL (top) and Spore Life (middle). Individuals can be in a dead (D), inactive (I) or active (A) state. Transitions among these states are governed by the number of active neighbours $N_{A}\in \{1,2,\ldots ,8\}$. Dormant individuals (i.e. spores) in an inactive state I die at every time step with probability 1−α (bottom). In (*b*), a selection of unique, repeating configurations $P_{n}$ with period n that arise in the deterministic limit (α=1) of Spore Life. Active individuals are shown in orange and inactive individuals in blue.

We developed cellular automata based on Conway’s GoL, which takes place on a two-dimensional lattice of sites S with periodic boundary conditions. In the original model, each site can be in a dead state (D) or occupied by an alive individual in a metabolically active state (A) such that S∈{active,dead}≡{A,D}. Initial conditions (t=0) are created by randomly seeding the ℓ×ℓ grid, where the probability q determines if sites are occupied by an active individual. In subsequent time steps, the grid is updated in a density-dependent manner. Specifically, the following rules are applied to the occupant of a focal site based on the number of active individuals ($N_{A}$) in neighbouring sites (figure 1):

- Active individual with <2 active neighbours dies (A→D)
- Active individual with 2−3 active neighbours persists (A→A)
- Active individual with >4 active neighbours dies (A→D)
- Dead individual with 3 active neighbours is reborn (D→A)

We then created a model called Spore Life by introducing dormancy into the cellular automaton. This required the addition of an inactive state (I), allowing individuals to be in one of three metabolic states: S∈{active,inactive,dead}≡{A,I,D}. As in the original GoL, initial conditions are established by seeding the ℓ×ℓ grid with a probability of q of each site being occupied by an active individual. In subsequent time steps, the grid is updated according to a slightly modified set of rules (figure 1):

- Active individual with 0 active neighbours dies (A→D)
- Active individual with 1 active neighbour becomes a dormant spore (A→I)
- Active individual with 2−3 active neighbours lives (A→A)
- Active individual with >4 active neighbours dies (A→D)
- Inactive individual (i.e. spore) with 2−3 active neighbours resuscitates (I→A)
- Dead individual with 3 active neighbours is reborn (D→A)

### (b) Spore survivorship

To explore how variation in spore survivorship influences population dynamics, we introduced a parameter α, which determines the fate of an inactive individual (figure 1*a*). We specify that an inactive individual dies with probability (1−α). As such, when α=1, an inactive individual has the potential to remain as a dormant spore for the remainder of the simulation. At the opposite limit, when α=0, all inactive individuals deterministically transition into dead individuals before the next time step. Under these conditions, the middle row of the Spore Life rule table is identical to the top row of the original GoL rule table (figure 1*a*). When α∈(0,1), transitions are stochastic such that some inactive individuals die while others resuscitate, reflecting variation in the degree to which organisms can persist in a shallow versus deep state of dormancy.

### (c) Population dynamics

Once implemented, we used Spore Life to characterize the effects of dormancy on key population-level phenomena. In addition to identifying the emergence of unique periodic configurations, we described how dormancy influences the abundance and distribution of metabolic states (A, I, D) on different-sized grids. We also measured extinction probabilities of the population. While controlling for starting densities across a broad range of initial condition (n = 1000), we quantified the number of time steps that occurred prior to extinction ($T_{\mathrm{ext}}$), which we operationally defined based on the constancy of $N_{\text{A}}$ after 100 time steps.

### (d) Demographic processes

To gain mechanistic insight into how dormancy affects population-level phenomena, we characterized two important demographic processes. First, we quantified the lifetime of inactive individuals for different levels of α. This involved calculating the number of time steps before an inactive spore either died or was resuscitated. We then compared these estimates to the lifetime of an active individual, which involved measuring the number of time steps before it either died or transitioned into an inactive state I. Second, we examined how α contributed to the population by comparing sources of births (i.e. D→A versus I→A) and sources of deaths (A→D versus A→I) as a function of α. An interactive web page is available to explore the dynamics of Spore Life: https://itp.uni-frankfurt.de/spore-life/

## 3. Results

### (a) Spore life creates unique periodic configurations

In the GoL (α=0) repeating configurations such as blinkers and gliders exist [28]. This also holds in the opposite limit (α=1) when Spore Life becomes deterministic. We identified a number of configuration that uniquely arise from the dormancy rule set (figure 1*a*). While our investigation of configurations was neither systematic nor exhaustive, we documented several patterns that repeat with periods of 2, 3, 4, 14 and 62 time steps (figure 1*b*; electronic supplementary material, figure S2).

### (b) Dormancy stabilizes population dynamics

**Figure 2.**
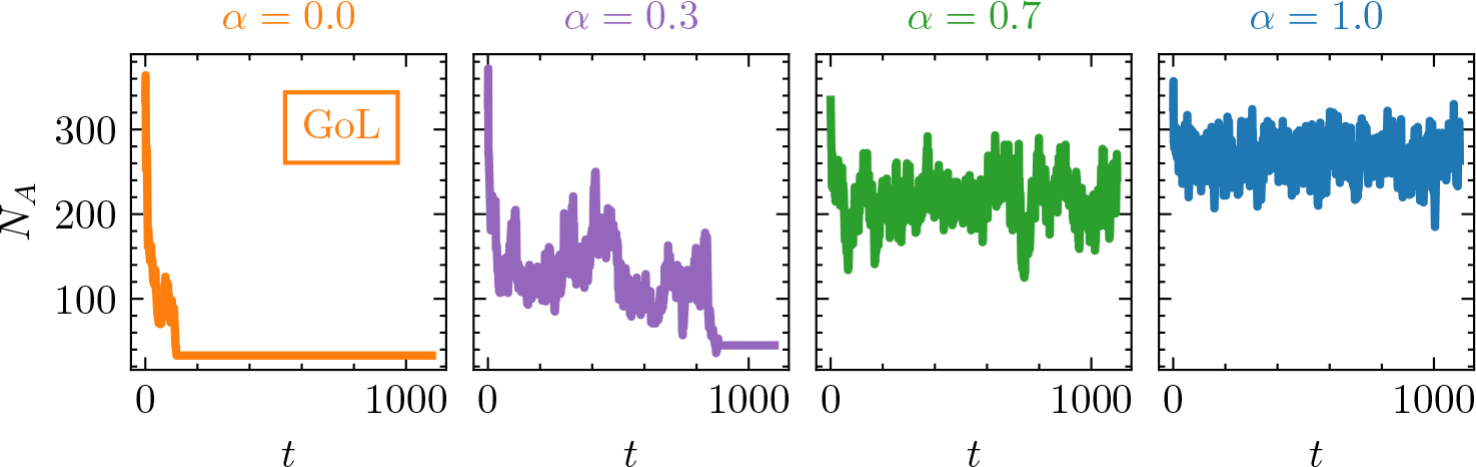
Population dynamics with dormancy. Abundance of active individuals ($N_{\text{A}}$) over time (t) for different levels of spore survivorship (α). For the GoL (α=0), the abundance of active individuals drops off rapidly to a small value $N_{\text{A}}/\ell^{2}\approx 0.033$, which is due to a limited number of surviving elementary configurations, mostly static or of period one. For a finite but low level of spore survivorship (α=0.3), $N_{\text{A}}$ is somewhat higher, but populations die out as a result of stochastic fluctuations. When spore survivorship is higher (α=0.7 and α=1), there is a corresponding increase in $N_{\text{A}}$. The grid size is ℓ×ℓ=30×30 with a starting density $\langle N_{A}\rangle /\ell^{2}=0.375$.

When starting from a random distribution of metabolic states, we find that Spore Life is much more stable than GoL (figure 2). Without dormancy (α=0), the number of active individuals ($N_{\text{A}}$) rapidly drops off leading to extinction in less than 100 time steps for a ℓ×ℓ=30×30 grid. Here, extinction occurs when there are no active or dormant individuals on the lattice or when the population is composed of isolated static or periodic configurations with densities of approximately3% active individuals. In contrast, when dormant spores were generated due to local-scale underpopulation, as illustrated in figure 1*a*, populations persist over prolonged time scales. In the deterministic limit, α=1, populations persisted for at least 1000 time steps with much higher average population sizes. A simulation comparing the dynamics of Spore Life and the GoL can be viewed at https://itp.uni-frankfurt.de/spore-life/resources/spore-life.gif. In between, for α∈(0,1), there was a reduction in the average $N_{\text{A}}$ relative to α=1, suggesting an increased risk of local extinction (electronic supplementary material, figure S1).

### (c) Diminished effect of dormancy in small populations

**Figure 3.**
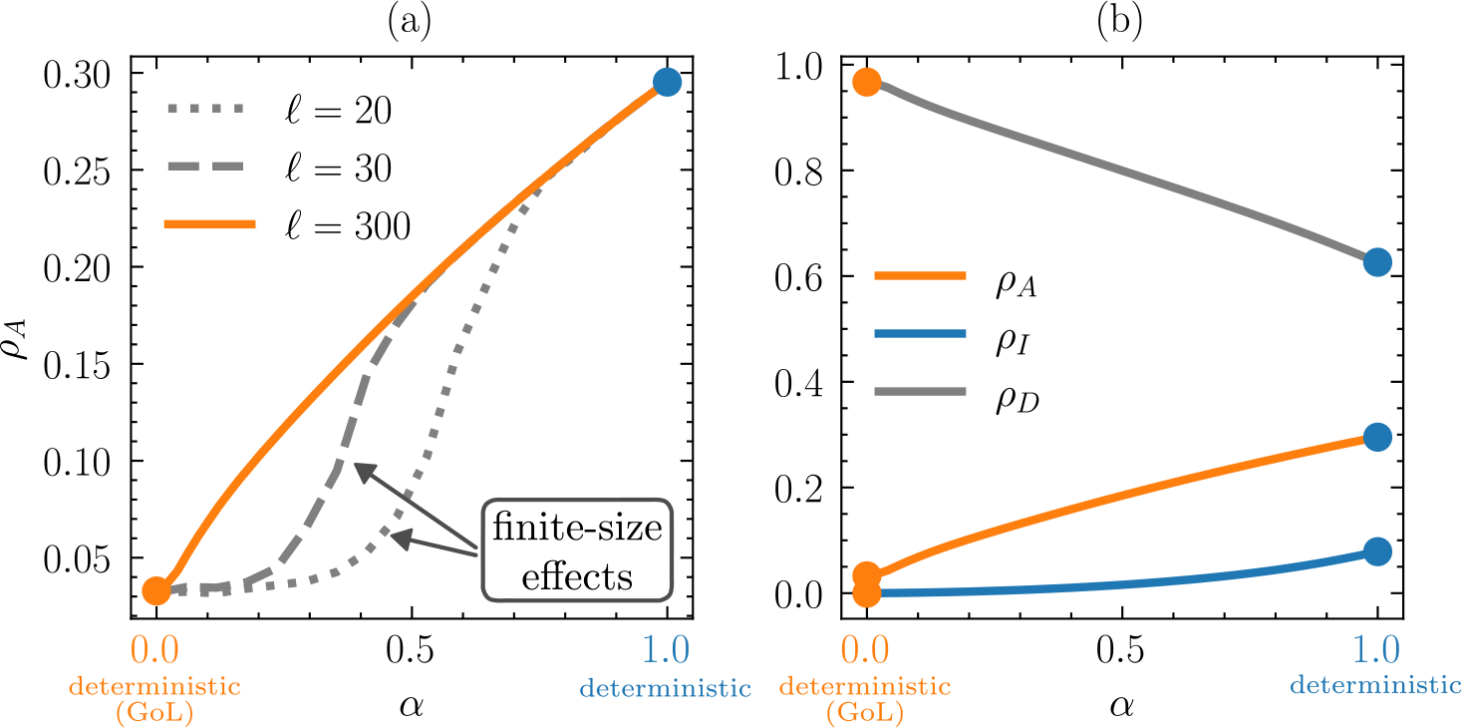
Finite-size effect of dormancy. In (*a*), the average density of active individuals $\rho_{\text{A}}=\langle N_{A}\rangle /\ell^{2}$ as a function of spore survivorship (α) on different-sized grids. For a larger grid (ℓ=300), the density of active individuals increases near linearly with α. The depression of $\rho_{\text{A}}$ on smaller grids (ℓ=20,30) represents a finite-size effect where small populations go extinct owing to stochastic fluctuations. Compare with figure 4 for further details. In (*b*), the average density of active ($\rho_{\text{A}}$), inactive ($\rho_{\text{I}}$) and dead ($\rho_{\text{D}}$) individuals is shown for ℓ=300.

We identify a finite-size effect of dormancy on population dynamics. When spore survivorship (α) is low, the stabilizing effect of dormancy on the average density of active individuals ($\rho_{\text{A}}=\langle N_{A}\rangle /\ell^{2}$) is weaker on smaller grids (e.g. ℓ=20 and ℓ=30) (figure 3*a*). Under such conditions, populations ($\left\langle N_{A}\right\rangle$) are smaller and experience larger fluctuations (electronic supplementary material, figure S2), increasing the probability of extinction because locally static or periodic configurations remain isolated for all time. On a larger grid (e.g. ℓ=300), however, the average density of active individuals increases near linearly with α (figure 3*b*). Under these conditions, where α≈1, the population is well mixed and individuals successively influence each other across all distances via the neighbourhood-based update rules (figure 1*a*).

**Figure 4.**
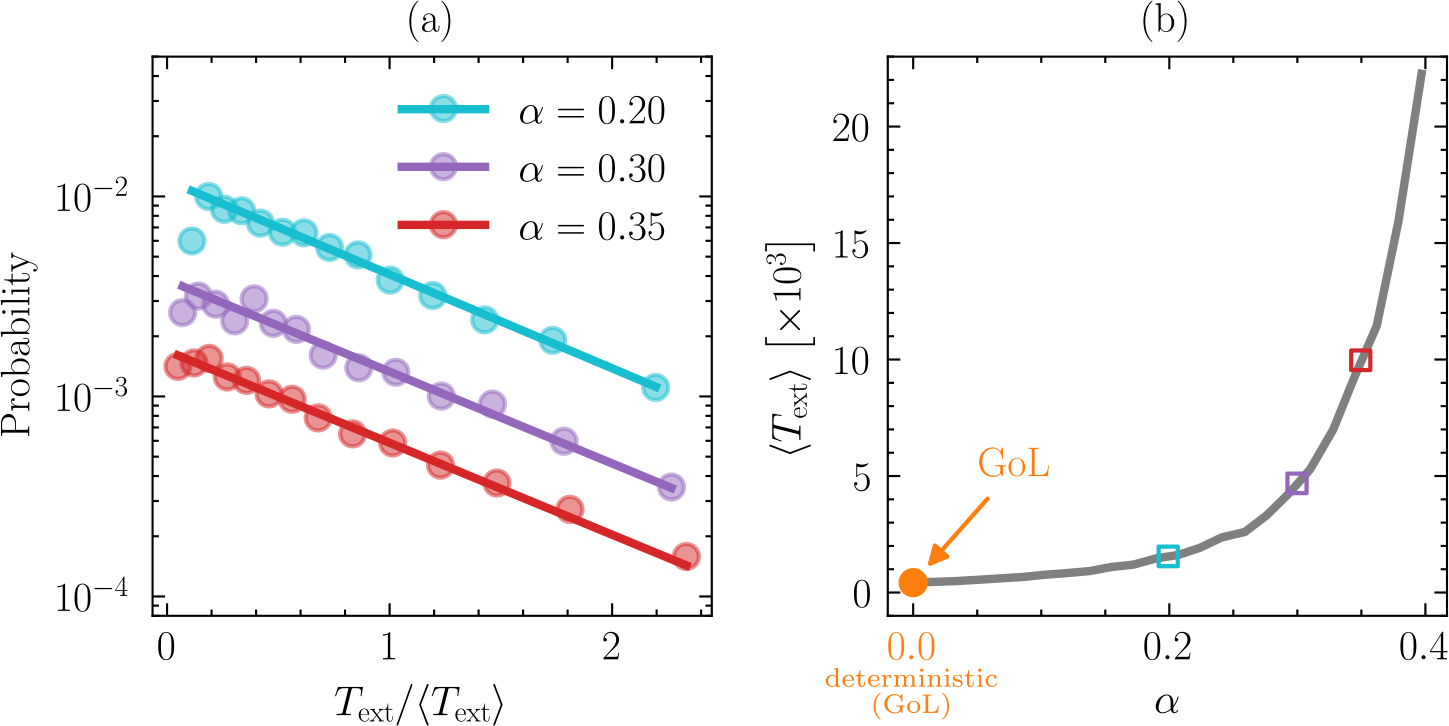
Extinction time increases with dormancy. (*a*) In Spore Life, we consider a population to be extinct if there are no active or dormant individuals, or if the pattern of active individuals consists only of isolated periodic or static configurations. From this, we define extinction time $T_{\mathrm{ext}}$ as the number of time steps starting from the random initial distribution of active individuals (t=0) until an extinct state is reached. For a ℓ×ℓ=30×30 grid, we averaged over $10^{3}$ initial conditions, finding that the distribution of extinction times $T_{\mathrm{ext}}$ is exponentially distributed. (*b*) On average, extinction times rise sharply with increasing spore survivorship α.

### (d) Dormancy alters the distribution of metabolic states

Spore survivorship alters the distribution of metabolic states in the population. Over the full range of α, dormancy leads to an increase of $\rho_{\text{A}}$ from ≈3% for α=0, to ≈30% at α=1 (figure 3*b*). At the same time, the average density of dead individuals ($\rho_{\text{D}}$) decreases by ≈35%. In contrast, the average density of inactive individuals ($\rho_{\text{I}}$) which can be viewed as the ‘seed bank’, remains low ($\rho_{\text{I}}<0.1$), even under maximal spore survivorship (α=1) (figure 3*b*).

### (e) Dormancy reduces extinction probability

Motivated by the temporal dynamics presented in figure 2, we sought to characterize the relationship between dormancy and the extinction probability. Considering a 30×30 grid, we found that extinction times ($T_{\mathrm{ext}}$) for a total of 1000 simulations were distributed according to an exponential law $∼c\;e^{-T_{\mathrm{ext}}/T}$ (figure 4*a*). We find $c\;[\times 10^{2}]=1.2/0.38/0.17$ and T=0.92/0.95/0.95 for α=0.2/0.3/0.35, respectively. While the decay constants (T) were essentially unaffected by spore survivorship (α), dormancy had a strong effect on the scaling factors (c), which is evident from the different intercepts in figure 4*a*. The average time to extinction $\left\langle T_{\mathrm{ext}}\right\rangle$ increases exponentially with increasing spore survivorship. In the absence of dormancy (α=0), $\left\langle T_{\mathrm{ext}}\right\rangle$ was ≤500 time steps, but even with moderate levels of spore survivorship (α=0.4), $\left\langle T_{\mathrm{ext}}\right\rangle$ already increased to ≥25000 time steps (figure 4*b*). Numerically, extinction times ($T_{\mathrm{ext}}$) increase with grid size.

### (f) Spore survivorship minimally extends spore lifetimes

To better understand how spore survivorship (α) influences population dynamics in Spore Life, we characterized the distribution of spore lifetimes. Once created, an inactive individual’s lifetime ($T_{\text{I}}$) is equal to the number of time steps until a mortality event (when α<1) or a resuscitation event occurs. The probability that an isolated spore remains present after t steps is


(3.1) $$\alpha^{t}=e^{t\log(\alpha )}=e^{-t/T_{\alpha }},\;T_{\alpha }=\frac{-1}{\log(\alpha )}\;,$$

indicating that the lifetime of inactive individuals is exponentially distributed.

**Figure 5.**
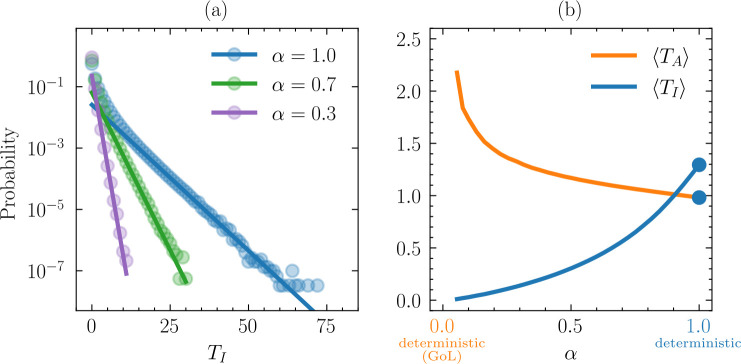
Distribution of spore lifetimes. (*a*) The time steps (T) that a dormant individual remains in an inactive state (*I*) before dying or resuscitating into an active state (A) decays exponentially. (*b*) The average life times of inactive individuals $\left\langle T_{\text{I}}\right\rangle$ and active individuals $\left\langle T_{\text{A}}\right\rangle$ as a function of α.

Owing to a sharp initial drop off (figure 5*a*), mean lifetimes are substantially shorter than the respective timescales (T) obtained through fits (figure 5*b*). Regardless of α, the average lifetime of a spore ($T_{\text{I}}$) is <1.5 time steps. Meanwhile, the average lifetime of an active individual ($T_{\text{A}}$) monotonically decreases with increasing α to about one time step for α→1 (figure 5*b*). Although we did not numerically explore the exact behaviour, it is worth noting that the lifetime of active individuals rapidly increases as α approaches 0. See electronic supplementary material for a discussion regarding the small values of α.

From the exponential distribution, we conclude that the natural decay of spores ∼(1−α) is the dominant cause for the loss of inactive individuals when α is not too close to one. By examining the tails of the spore lifetime distributions (figure 5*a*), which can be approximated by an exponential ∼exp⁡(−t/T), we find T=0.77/2.14/4.56 for α=0.3/0.7/1.0, whereas equation (3.1) would predict $T_{\alpha }=0.82/2.80/\infty$, which is in turn reasonably close for α=0.3 and α=0.7. On the other hand, inactive individuals can only change to active individuals when α=1, a process for which the typical timescale is observed to be T=4.56.

### (g) Spore survival alters demographic processes

**Figure 6.**
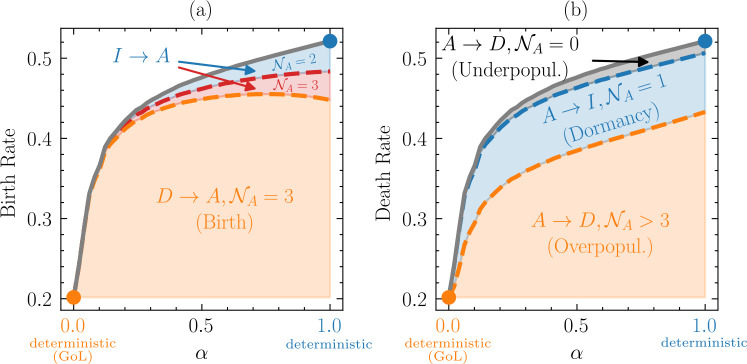
Influence of dormancy on demographic processes. The gray lines correspond to (*a*) the birth rate and (*b*) the death rate of active individuals (A) as a function of spore survivorship (α). The birth rate is defined as the number of transitions to the active state (D→A or I→A) per time step normalized by the average number of active individuals $\left\langle N_{A}\right\rangle$, that is $Birth\;Rate=(\#\text{transitions to active})/\langle N_{A}\rangle$. The death rate is defined as the number of transitions out of the active state (A→D or A→I) per time step normalized by $\left\langle N_{A}\right\rangle$. For the birth rate, the primary source of new individuals comes from D→A transitions. Resuscitation events (I→A) from individuals surrounded by either 2 or 3 active neighbours contribute equally, but rather minimally to the overall birth rate, especially at lower α. For the death rate, the most dominant cause of death is overcrowding (A→D when ≥3 active neighbours), while the contribution of underpopulation (A→D for 0 active neighbours) is relatively small. Dormancy (A→I) plays an increasingly important role with increasing spore survivorship (α).

Spore survivorship (α) substantially affects the birth rates and death rates of active individuals (figure 6). For small α, about 20% of the population dies and is reborn at every time step. As α→1, ≥50% of the population dies and is reborn at every step. This is due to a higher likelihood of mortality from overcrowding (figure 6*b*) with increasing population density (figure 3). Somewhat counterintuitively, the probability of an active individual transitioning into the inactive state (I) increases substantially over the same range of α.

Regarding birth processes, at low to intermediate levels of α, the vast majority of births (≥90%) occur when active individuals are produced in an empty (dead) site that was surrounded by three active neighbours. As α→1, about 13% of new births are associated with the transition from an inactive state (I) to an active (A) state. As illustrated in figure 1*a*, these resuscitations happen when a spore is surrounded by either 2 or 3 active neighbours ($N_{A}\in \{2,3\}$). Our analysis suggests that the contributions of these two resuscitation pathways to the population birth rate are roughly equivalent (figure 6*a*). Regardless of whether an individual is in a dead (D) or inactive (I) state, when $N_{A}=3$, an active individual will be born. This means that any birth-mediated effect of dormancy can be attributed to the metabolic transitions that occur when $N_{A}=2$, which never accounts for more than ≈8% of the birth rate (figure 6*a*). Instead, at least 88% of all new births are associated with D→A transitions, even when α=1.

Similarly, we analysed how different metabolic transitions contributed to the death rate of a population as a function of spore survival (α). The largest source of mortality (≈80%) is due to overpopulation, which occurs when an individual is surrounded by ≥4 active neighbours (figure 6b). In contrast, underpopulation is the smallest source of mortality (≈2%), which occurs when an active individual is surrounded by 0 active neighbours. Last, ≥18% of the death rate was due to the loss of active individuals that transitioned into an inactive state (I) when they were surrounded by 1 active individual.

## 4. Discussion

Minor modifications to a simple cellular automaton successfully captured critical population-level phenomena commonly associated with dormancy. Using Spore Life, we demonstrate that dormancy confers stability, defined as population persistence over time (figure 2, electronic supplementary material, figure S1). To explore the effects of shallow versus deep dormancy, we included a spore survivorship parameter (α) that also introduces stochasticity into the model. As expected, extinction times increase with higher spore survivorship (figure 4). Unexpectedly, the benefits of dormancy do not require the accumulation of inactive individuals into a large or long-lived seed bank. Instead, dormancy promotes population persistence through slight increases in spore lifetimes (figure 5), modest contributions to demographic rates (figure 6) and feedbacks involving spatial processes (electronic supplementary material, figure S3) along with the emergence of unique periodic configurations (figure 1*b*, electronic supplementary material, figure S2). Our findings reveal that small changes to a relatively simple cellular automaton can yield complex behaviours that are consistent with theoretical expectations of how dormancy stabilizes populations in fluctuating environments. In the following sections, we discuss these findings in the context of existing knowledge about dormancy from other computational models. We also explore how our results can guide future research efforts, including empirical studies related to the resilience of host and environmental health.

### (a) Scale-dependence of dormancy

Dormancy is governed by a range of distinct processes that operate across spatial and temporal scales [3]. Some drivers of dormancy (e.g. photoperiod) occur seasonally and span large geographic extents [29]. Dormancy can also be triggered by local conditions, including density-dependent fluctuations in the abundance of individuals within a cohort that experience intraspecific competition for a limiting resource [30]. In Spore Life, dormancy dynamics are entirely controlled by the metabolic transitions of individuals and their nearest neighbors, rather than external forces. These local interactions play out across the entire landscape, determining the emergent dynamics of the population.

One scale-dependent phenomenon observed in our study relates to the effect of dormancy in habitats (i.e. grids) of different sizes. In GoL, which lacks dormancy (α=0), the proportion of active individuals ($\rho_{\text{A}}$) rapidly declines over time for the initial conditions considered in our study (figure 2). Consistent with an Allee effect [31], a critical point is reached where local neighbourhoods are so depleted of active individuals that new individuals cannot be born (D→A), leading to the effective extinction of the population. In Spore Life, where α>0, the opposite is true, especially when α is large (α→1). The proportion of active individuals ($\rho_{\text{A}}$) is sustained at a much higher level (figure 2) owing to the dormancy refuge (A→I) and resuscitation events (I→A). In the two deterministic limits (α=0 and α=1), the effects of dormancy are consistent across simulations, regardless of grid size. However, at intermediate levels of spore survivorship, the effects of dormancy are more variable. Specifically, on small grids with low to intermediate levels of α, there is a higher probability that elementary configurations become fixed, leading to low $N_{\text{A}}$ and subsequent extinction. In contrast, populations persist longer with low to intermediate levels of α on larger grids. This finite-size effect may reflect unique features of our cellular automaton. Alternatively, the model may be capturing a more general phenomenon—albeit one that is not well-documented—where smaller populations, particularly in fragmented habitats, are less likely to be rescued by dormancy.

### (b) Shallow versus deep dormancy

Dormancy is a process that can lead to the formation of a seed bank, a subpopulation of metabolically inactive individuals. There are many seed-bank attributes that can affect the ecology and evolution of a population [7]. One important attribute is seed bank size. A large seed bank, composed of many inactive individuals, provides more opportunities for resuscitating individuals, thereby buffering a population against extinction. Another important attribute is seed bank turnover, which is influenced by the duration that dormant individuals can survive in a metabolically inactive state. Longer-lived dormant individuals can provide greater insurance to a population in a fluctuating environment, as long as they remain capable of resuscitation. However, prolonged dormancy can be maladaptive because it delays growth and reproduction [32]. These general hypotheses led us to introduce the parameter α into our model to explore how spore survivorship influences the outcomes of dormancy. We found that the effects of dormancy on the population were significantly affected by α. Specifically, the abundance of active individuals ($N_{\text{A}}$) increases with α, and the probability of extinction decreases with α.

Contrary to our expectations, spore survivorship (α) did not have a strong effect on seed bank size (i.e. $\rho_{\text{I}}$), or on the lifetime of a spore ($T_{\text{I}}$). Instead, the effects of dormancy were achieved by individuals that effectively only spent a short time in an inactive state (I). The average lifetime of a spore was quite low, on the order of only a single time step (or generation). Meanwhile, the contribution of resuscitation (I→A) to the overall birth rate of active individuals was low (<12%) compared to births not directly associated with dormancy (D→A). On the larger grids (e.g. 300×300), the density of inactive individuals ($\rho_{\text{I}}$) never comprised more than 8% of the grid or 22% of the viable (i.e. active+inactive) individuals. Seed bank size, defined as $N_{\text{I}}$ : $N_{\text{A}}$, increased with α, but was generally low (0.02–0.27). While turnover increased with α, resuscitation with $N_{A}=2$ living neighbours never accounted for >11% of all births.

One explanation for population persistence in Spore Life (figure 2) is sustained chaotic transients. In complex systems, the introduction of noise can lead to superpersistent transient chaos [33]. Specifically, lattice-wide noise could be generated in simulations with stochastic spore survivorship (α∈(0,1)). However, this explanation does not align with the observed behaviour in the deterministic limit of Spore Life (α=1), where we see clear stabilization of populations induced by dormancy.

Another possibility is that dormancy may be crucial for population persistence, even if inactive individuals are short-lived and seed banks are relatively small. Such ideas are not well developed in theoretical treatments of dormancy, although biologists have observed ‘cryptic’ forms of metabolic stasis in certain taxa [34,35]. Our findings suggest there may be relatively unexplored interacting processes and regimes where dormancy is important for stabilizing populations, necessitating the simultaneous development of theoretical and empirical approaches.

### (c) Dormancy-mediated spatial processes contribute to population persistence

Although dormancy is typically viewed as a temporal phenomenon, it is increasingly recognized for its influence on spatial processes and patterns [36]. In Spore Life, when α > 0, we noted that small, isolated patches of individuals tend to survive, expand and eventually repopulate the grid (see animated simulation: https://doi.org/10.6084/m9.figshare.27239748).

This observation led us to hypothesize that dormancy enhances population persistence by influencing spatial processes. To test this, we used a mean-field theory approach where the transition between metabolic states follow the rules in figure 1, but without the spatial structure imposed by the Spore Life lattice (see electronic supplementary material). In the absence of spatial structure, persistence is sensitive to initial conditions. Specifically, when the proportion of active individuals ($\rho_{\text{A}}$) is low, the population goes extinct (electronic supplementary material, figure S3). This behaviour is opposite of what we observe in Spore Life (α > 0), suggesting that spatial processes play an important mechanistic role in dormancy-mediated rescue of small populations from extinction.

While the strength of spores lies in their population-spreading capacity, a distinct dormancy effect also plays a crucial role in enhancing persistence. We observe that spore survivorship (α) reduces the proportion of active individuals ($\rho_{\text{A}}$) that are required for persistence while simultaneously increasing the asymptotic proportion of active individuals ($\rho_{\text{A}}$) in the population. However, this effect of dormancy in the mean field is dependent on neighbourhood size ($N_{neighbours}$). In populations with a smaller neighbourhood size ($N_{neighbours}=6$), spore survivorship (α) significantly reduces the proportion of active individuals needed for persistence. In contrast, when the neighbourhood size is larger ($N_{neighbours}=10$), the effects of dormancy are nearly eliminated (electronic supplementary material, figure S3). The metabolic transition rules (figure 1) were specifically designed for a lattice-based configuration with $N_{neighbours}=8$, so it is unsurprising that population dynamics shift with changes in $N_{neighbours}$. Nevertheless, these findings raise intriguing questions for future research, particularly regarding the role of dormancy in systems with varying degrees of dispersion and overall population size (N).

### (d) How ‘easy’ is dormancy?

Dormancy has evolved independently numerous times across the tree of life, making it impossible to create a single model that represents all features and attributes of this diverse life-history strategy. Therefore, our goal was to develop a compact model with the fewest possible rules and assumptions. We find that Spore Life successfully recapitulates a range of canonical behaviours associated with dormancy, including the dampening of population dynamics in fluctuating environments [37].

That being said, there are additional seed-bank attributes that could be incorporated into future versions of Spore Life. In nature, dormant and active individuals are typically not well mixed [7]. For instance, actively growing parts of a plant are usually found aboveground, while dormant seeds, after dispersing from their natal sites, often reside belowground in the soil. This population structure could be represented in the metabolic transition rules of Spore Life (figure 1*a*). Additionally, coarser-grained heterogeneity could be encoded onto a grid, or even a three-dimensional spatial lattice of a cellular automaton [38,39], enabling tests of seed bank dynamics in a more complex universe. One might expect that the spatial decoupling of dormant and active individuals would reduce the system’s reactivity, giving rise to larger and longer-lived seed banks. Such questions should also be explored using alternative modeling frameworks, such as ordinary differential equations, to offer complementary insights and enhance generality.

Shifts in organismal metabolism can be influenced by various factors. In the current version of Spore Life, we primarily examine conditions where dormancy is triggered by underpopulation (figure 1*a*), specifically when an active individual is surrounded by only one other active individual $N_{A}\in \{1\}$). This rule is designed to represent the metabolic changes that occur when local population densities decrease due to a decline in environmental quality. Of course, important demographic processes are also affected by overpopulation, some of which, like logistic growth, have been incorporated into GoL models [40]. Dormancy can also be induced by crowding [41], including among kin [42], leading to resource depletion, growth inhibition from waste byproducts, or the buildup of parasites. Therefore, overpopulation represents another pathway that can initiate dormancy. As a validation step, we implemented a modified version of Spore Life where the transition from an active (A) to an inactive state (I) occurs when $N_{A}=4$. Although this rule adjustment led to changes in absolute densities, the qualitative effects on population dynamics remained consistent, suggesting that our findings are robust to different implementations of dormancy.

Last, while we incorporated stochasticity in the context of spore survivorship, further investigation is needed to understand how population dynamics are shaped by the interplay between random and deterministic regulation of metabolic transitions [43]. Such efforts would facilitate exploration and synergy with other areas of dormancy-related research, including evolutionary adaptive dynamics [44], ecological responses to climate change [45], interacting particle systems in statistical physics [46] and the treatment of diseases such as chronic infections [47] and cancer [48]. Ultimately, the platform developed here has the potential to illuminate the origins and diversification of a widespread trait characterized by spatial and temporal dynamics [18], along with the emergence of complex behaviours associated with metabolic feedback loops.

## Data Availability

Code for the model and analyses is available on GitHub [49] and Figshare [50]. An animated simulation has been uploaded to FigShare. Supplementary material is available online [51].

## References

[1] Parmesan C. 2006 Ecological and evolutionary responses to recent climate change. Annu. Rev. Ecol. Evol. Syst. **37**, 637–669. (10.1146/annurev.ecolsys.37.091305.110100)

[2] Burton T, Ratikainen II, Einum S. 2022 Environmental change and the rate of phenotypic plasticity. Glob. Chang. Biol. **28**, 5337–5345. (10.1111/gcb.16291) 35729070 PMC9541213

[3] Jones SE, Lennon JT. 2010 Dormancy contributes to the maintenance of microbial diversity. Proc. Natl Acad. Sci. USA **107**, 5881–5886. (10.1073/pnas.0912765107) 20231463 PMC2851880

[4] Gerber N, Kokko H. 2018 Abandoning the ship using sex, dispersal or dormancy: multiple escape routes from challenging conditions. Phil. Trans. R. Soc. B **373**, 20170424. (10.1098/rstb.2017.0424) 30150222 PMC6125733

[5] Sorensen JW, Shade A. 2020 Dormancy dynamics and dispersal contribute to soil microbiome resilience. Philos. Trans. R. Soc. Lond., B, Biol. Sci. **375**, 20190255. (10.1098/rstb.2019.0255) 32200738 PMC7133531

[6] Menges ES. 2000 Population viability analyses in plants: challenges and opportunities. Trends Ecol. Evol. **15**, 51–56. (10.1016/s0169-5347(99)01763-2) 10652555

[7] Lennon JT, den Hollander F, Wilke-Berenguer M, Blath J. 2021 Principles of seed banks and the emergence of complexity from dormancy. Nat. Commun. **12**. (10.1038/s41467-021-24733-1) PMC8355185 34376641

[8] McKenney PT, Driks A, Eichenberger P. 2013 The Bacillus subtilis endospore: assembly and functions of the multilayered coat. Nat. Rev. Microbiol. **11**, 33–44. (10.1038/nrmicro2921) 23202530 PMC9910062

[9] Kikuchi K, Galera-Laporta L, Weatherwax C, Lam JY, Moon EC, Theodorakis EA, Garcia-Ojalvo J, Süel GM. 2022 Electrochemical potential enables dormant spores to integrate environmental signals. Science **378**, 43–49. (10.1126/science.abl7484) 36201591 PMC10593254

[10] Kussell E, Leibler S. 2005 Phenotypic diversity, population growth, and information in fluctuating environments. Science **309**, 2075–2078. (10.1126/science.1114383) 16123265

[11] Wright ES, Vetsigian KH. 2019 Stochastic exits from dormancy give rise to heavy‐tailed distributions of descendants in bacterial populations. Mol. Ecol. **28**, 3915–3928. (10.1111/mec.15200) 31355980

[12] Wilsterman K, Ballinger MA, Williams CM. 2021 A unifying, eco‐physiological framework for animal dormancy. Funct. Ecol. **35**, 11–31. (10.1111/1365-2435.13718)

[13] Brenner EV *et al*. 2013 Draft genome sequence of Bacillus cereus strain F, isolated from ancient permafrost. Genome Announc. **1**. (10.1128/genomeA.00561-13) PMC3731846 23908292

[14] Willis CG, Baskin CC, Baskin JM, Auld JR, Venable DL, Cavender-Bares J, Donohue K, Rubio de Casas R, Grp NEGW. 2014 The evolution of seed dormancy: environmental cues, evolutionary hubs, and diversification of the seed plants. New Phytol. **203**, 300–309. (10.1111/nph.12782) 24684268

[15] Cohen D. 1966 Optimizing reproduction in a randomly varying environment. J. Theor. Biol. **12**, 119–129. (10.1016/0022-5193(66)90188-3) 6015423

[16] Ten Brink H, Gremer JR, Kokko H. 2020 Optimal germination timing in unpredictable environments: the importance of dormancy for both among‐ and within‐season variation. Ecol. Lett. **23**, 620–630. (10.1111/ele.13461) 31994356 PMC7079161

[17] Măgălie A, Schwartz DA, Lennon JT, Weitz JS. 2023 Optimal dormancy strategies in fluctuating environments given delays in phenotypic switching. J. Theor. Biol. **561**, 111413. (10.1016/j.jtbi.2023.111413) 36639023

[18] Webster KD, Lennon JT. 2025 Dormancy in the origin, evolution and persistence of life on Earth. Proc. R. Soc. B **292**, 20242035. (10.1098/rspb.2024.2035) PMC11706647 39772956

[19] Gardner M. 1970 Mathematical games: the fantastic combinations of John Conway’s new solitaire game 'life'. Sci. Am. **223**, 120–123. (10.1038/scientificamerican1070-120)

[20] Gotts NM. 2000 Emergent phenomena in large sparse random arrays of Conway’s ‘Game of Life’. Int. J. Syst. Sci. **31**, 873–894. (10.1080/002077200406598)

[21] Nowak MA, May RM. 1992 Evolutionary games and spatial chaos. Nature **359**, 826–829. (10.1038/359826a0)

[22] Bak P, Chen K, Creutz M. 1989 Self-organized criticality in the ‘Game of Life’. Nature **342**, 780–782. (10.1038/342780a0)

[23] Beer RD. 2015 Characterizing autopoiesis in the Game of Life. Artif. Life **21**, 1–19. (10.1162/ARTL_a_00143) 25148547

[24] El Yacoubi S, El Jai A. 2002 Cellular automata modelling and spreadability. Math. Comput. Model. **36**, 1059–1074. (10.1016/s0895-7177(02)00259-5)

[25] Hwang M, Garbey M, Berceli SA, Tran-Son-Tay R. 2009 Rule-based simulation of multi-cellular biological systems—a review of modeling techniques. Cell. Mol. Bioeng. **2**, 285–294. (10.1007/s12195-009-0078-2) 21369345 PMC3045734

[26] Javid MAJ, te Boekhorst R. 2006 Cell dormancy in cellular automata. In Computational science—ICCS 2006 (eds VN Alexandrov, GD van Albada, PMA Sloot, J Dongarra), pp. 367–374. Berlin, Germany: Springer. (10.1007/11758532_50)

[27] Locey KJ, Fisk MC, Lennon JT. 2016 Microscale insight into microbial seed banks. Front. Microbiol. **7**, 2040. (10.3389/fmicb.2016.02040) 28119666 PMC5220057

[28] Brown N, Cheng C, Jacobi T, Karpovich M, Merzenich M, Raucci D, Riley M. 2023 Conway’s Game of Life is omniperiodic. arXiv (10.48550/arXiv.2312.02799)

[29] Saunders DS. 2020 Dormancy, diapause, and the role of the circadian system in insect photoperiodism. Annu. Rev. Entomol. **65**, 373–389. (10.1146/annurev-ento-011019-025116) 31594413

[30] Nilsson P, Tuomi J, Fagerstrom T, Astrom M. 1994 Does seed dormancy benefit the mother plant by reducing sib competition? Evol. Ecol. **8**, 422–430. (10.1007/BF01238192)

[31] Stephens PA, Sutherland WJ, Freckleton RP. 1999 What Is the Allee effect? Oikos **87**, 185. (10.2307/3547011)

[32] Gremer JR, Crone EE, Lesica P. 2012 Are dormant plants hedging their bets? Demographic consequences of prolonged dormancy in variable environments. Am. Nat. **179**, 315–327. (10.1086/664459) 22322220

[33] Do Y, Lai YC. 2005 Scaling laws for noise-induced superpersistent chaotic transients. Phys. Rev. E Stat. Nonlinear Soft Matter Phys. **71**, 046208. (10.1103/PhysRevE.71.046208) 15903771

[34] Doropoulos C, Bozec YM, Gouezo M, Priest MA, Thomson DP, Mumby PJ, Roff G. 2022 Cryptic coral recruits as dormant 'seed banks': an unrecognized mechanism of rapid reef recovery. Ecology **103**, e3621. (10.1002/ecy.3621) 34939185

[35] Polačik M, Vrtílek M. 2023 Cryptic stasis during the development of Nothobranchius furzeri suggests new stages of dormancy outside of the typical three diapauses of annual killifishes. Environ. Biol. Fishes **106**, 575–583. (10.1007/s10641-023-01393-2)

[36] Wisnoski NI, Leibold MA, Lennon JT. 2019 Dormancy in metacommunities. Am. Nat. **194**, 135–151. (10.1086/704168) 31318286

[37] Cáceres CE. 1997 Temporal variation, dormancy, and coexistence: a field test of the storage effect. Proc. Natl Acad. Sci. USA **94**, 9171–9175. (10.1073/pnas.94.17.9171) 11038565 PMC23092

[38] Adamatzky A. 2010 Game of life cellular automata. Berlin, Germany: Springer. (10.1007/978-1-84996-217-9)

[39] Glade N, Bastien O, Ballet P. 2017 Diversity and survival of artificial lifeforms under sedimentation and random motion. Theory Biosci. **136**, 153–167. (10.1007/s12064-017-0254-1) 28721495

[40] Kawano T. 2020 Translating the Conway’s Game of Life as a discrete logistic cellular automata model with density. Effects. Int. J. Innov. Comput. Inf. Cont. **16**, 1655–1666.

[41] Habtewold T, Sharma AA, Wyer CAS, Masters EKG, Windbichler N, Christophides GK. 2021 Plasmodium oocysts respond with dormancy to crowding and nutritional stress. Sci. Rep. **11**, 3090. (10.1038/s41598-021-81574-0) 33542254 PMC7862253

[42] Twyman KZ, Gardner A. 2023 Kin selection of time travel: the social evolutionary causes and consequences of dormancy. Proc. R. Soc. B **290**, 20231247. (10.1098/rspb.2023.1247) PMC10498053 37700652

[43] Blath J, Tóbiás A. 2020 Invasion and fixation of microbial dormancy traits under competitive pressure. Stoch. Process. Their Appl. **130**, 7363–7395. (10.1016/j.spa.2020.07.018)

[44] Blath J, Paul T, Tóbiás A. 2023 A stochastic adaptive dynamics model for bacterial populations with mutation, dormancy and transfer. Lat. Am. J. Probab. Math. Stat. **20**, 313. (10.30757/alea.v20-12)

[45] Ooi MKJ, Auld TD, Denham AJ. 2009 Climate change and bet‐hedging: interactions between increased soil temperatures and seed bank persistence. Glob. Chang. Biol. **15**, 2375–2386. (10.1111/j.1365-2486.2009.01887.x)

[46] Floreani S, Giardinà C, Hollander F den, Nandan S, Redig F. 2022 Switching Interacting particle systems: scaling limits, uphill diffusion and boundary layer. J. Stat. Phys. **186**. (10.1007/s10955-022-02878-7)

[47] Rittershaus ESC, Baek SH, Sassetti CM. 2013 The normalcy of dormancy: common themes in microbial quiescence. Cell Host Microbe **13**, 643–651. (10.1016/j.chom.2013.05.012) 23768489 PMC3743100

[48] Aguirre-Ghiso JA. 2007 Models, mechanisms and clinical evidence for cancer dormancy. Nat. Rev. Cancer **7**, 834–846. (10.1038/nrc2256) 17957189 PMC2519109

[49] Nevermann DH. 2024 spore-life. See https://github.com/ISCOTTYI/spore-life.

[50] Nevermann DH, Gros C, Lennon JT. 2024 Code for: A Game of Life with dormancy. Figshare. (10.6084/m9.figshare.27952776) PMC11775590 39876717

[51] Nevermann DH, Gros C, Lennon JT. 2025 Supplementary material from: A Game of Life with dormancy. Figshare (10.6084/m9.figshare.c.7634133) PMC11775590 39876717

